# Addressing gender disparities in eye health services among school-age girls in India

**Published:** 2025-03-07

**Authors:** Meenakshi Shah, Surender Kumar, Ananta Basudev Sahu

**Affiliations:** 1 Programme Officer, Quality: Sightsavers India, New Delhi.; 2 Manager, Programme: Sightsavers India, New Delhi.; 3 Senior Manager, Programme Performance Research and Learning: Sightsavers India, New Delhi.

**By emphasising community engagement and gender inclusivity, a school eye programme was able to improve access to eye care for girls.**

**Figure:**
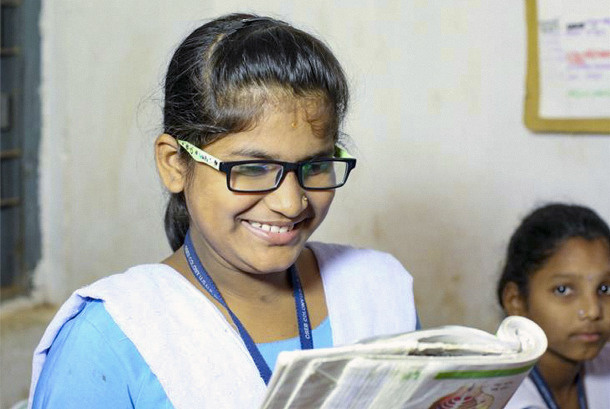
Sitara, a 12-year-old girl from an intervention district in Madhya Pradesh, struggled academically due to undiagnosed vision problems, which affected her ability to see the blackboard and read textbooks. During a school eye screening camp, her impairment was identified, and she was referred for a comprehensive eye examination. With her new glasses, Sitara's confidence and academic performance improved. india

© SIGHTSAVERS INDIA CC BY-NC-SA 4.0

With nearly 29% of its population under the age of 15, India is home to an estimated 33.4 million children who require spectacles for vision correction. However, many children, particularly girls, remain underserved.^1,^^2^ A study conducted in Telangana, India found that girls are at greater risk of developing myopia, with an adjusted odds ratio of 1.30 compared to boys. Effective refractive error coverage for girls (39.06%) lags behind that of boys (47.04%).^3,^^4^ India's National Programme for Control of Blindness and Visual Impairment (NPCBVI) has made significant strides in addressing childhood visual impairment through initiatives such as the School Eye Screening Programme, which trains teachers to carry out preliminary vision screenings and referrals.^5,^^6^ However, challenges remain, including limited reach, inadequate follow-up, and a lack of gender-sensitive strategies, disproportionately affecting girls in underserved areas.

The Vidyajyoti School Eye Health Programme, by Sightsavers India, seeks to bridge the gap in eye health services for children in government schools by conducting comprehensive screenings, implementing preventive strategies, and adopting an inclusive approach focused on equity and accessibility. Under the programme, over 362,000 students have been screened (52% of whom are girls). A total of 17,107 children requiring spectacles have been identified (58% of whom are girls, with 63% of these girls receiving spectacles). In total, 14,528 children have received spectacles.

## Strategies

The programme strategies are as follows:

- **Awareness campaigns.** Raising community awareness about the importance of eye health; educating students, particularly girls, about the benefits of wearing spectacles and dispelling myths associated with vision correction
- **Parental engagement.** Encouraging mothers’ involvement in eye health discussions, as maternal literacy plays a crucial role in ensuring girls’ compliance with wearing spectacles
- **Peer support groups.** Facilitating groups where girls can share experiences and encourage each other to wear spectacles, reducing stigma and fostering compliance
- **Addressing operational challenges.** Tackling systemic issues such as school absenteeism, inadequate infrastructure, and delays in delivery of spectacles
- **Gender- and culture-sensitivity training.** Training teachers and health educators to address gender gaps, harmful gender norms and roles, and socio-cultural barriers
- **Improving infrastructure.** Enhancing school infrastructure to support effective screening
- **Community-based outreach programmes.** Provision of eye care services, particularly in rural areas, which indirectly benefits women and girls by enhancing overall access to eye health services
- **Collaboration with NGOs and the private sector.** Improving resource availability and support for eye health initiatives, thus creating an inclusive environment for women and girls.

Sustainable change requires integrating strategies into broader policies, embedding gender equity in programmes like NPCBVI, and ensuring funding and accountability. Using sex-disaggregated data to understand the gender gap and forming deliberate collaborations among educators, health care educators, and community organisations can help close the gender gap in refractive error coverage. This will empower children—especially girls—to excel academically and socially, ensuring a healthier future in alignment with the Sustainable Development Goals.

## References

[1] Government of India, Ministry of Health and Family Welfare. National health profile 2015..

[2] Dandona R., Dandona L. (2001). Refractive error blindness.. Bulletin World Health Organ..

[3] Prakash WD., Marmamula S., Keeffe J., Khanna RC. (2024). Effective refractive error coverage and spectacle coverage among school children in Telangana, South India.. Eye..

[4] Impact and Policy Research Institute. School screenings and eye care [Internet].. https://tinyurl.com/yywdzty9.

[5] Rustagi N., Uppal Y., Taneja DK. (2012). Screening for visual impairment: outcome among schoolchildren in a rural area of Delhi.. Indian J Ophthalmol..

[6] Murthy GVS. (2000). Vision testing for refractive errors in schools: ‘screening’ programmes in schools.. Community Eye Health..

